# Supplementary material for: Asymmetric Synthesis of Quaternary Hydantoins via a Palladium-Catalyzed Aza-Heck Cyclization
Source: J Am Chem Soc. 2025 Nov 14;147(49):44692–8. doi: 10.1021/jacs.5c16022 (PMC12703750; doi:10.1021/jacs.5c16022)
Supplement: Supplementary file 2 [file ja5c16022_si_002.zip › All NMR FID Files/S56/S56_ProtonNMR/2/pdata/1/email_MLM03073_2_1.pdf]

C13CPD1024 CDCl3 /opt/nmrdata mlmurr 13

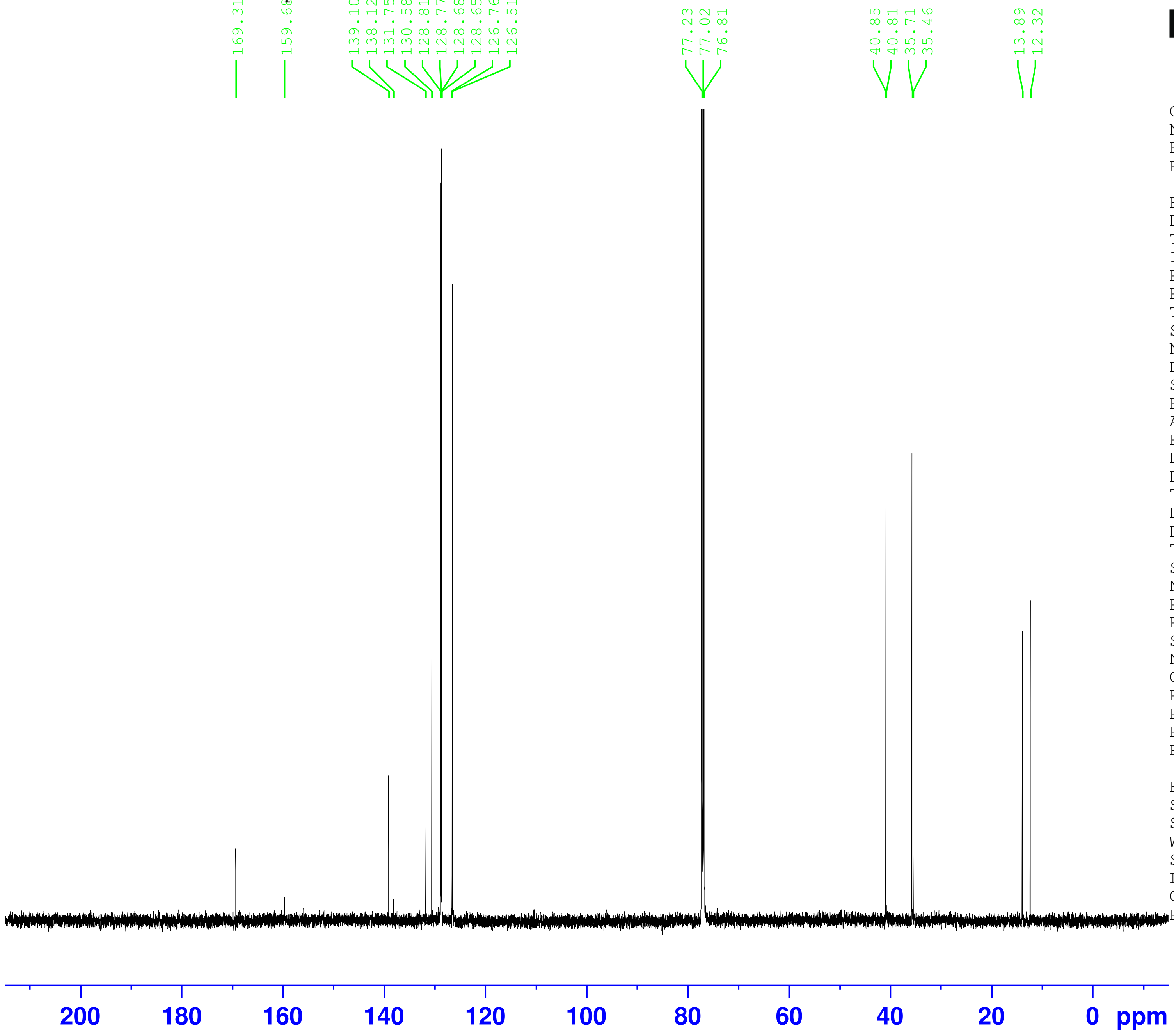

Current Data Parameters

NAME	MLM03073
EXPNO	2
PROCNO	1

F2 - Acquisition Parameters

Date_	20240613
Time	18.18 h
INSTRUM	spect
PROBHD	Z168773_0053 (
PULPROG	zgpg55
TD	65536
SOLVENT	CDCl3
NS	1024
DS	4
SWH	34722.223 Hz
FIDRES	1.059638 Hz
AQ	0.9437184 sec
RG	1030
DW	14.400 usec
DE	19.47 usec
TE	298.0 K
D1	1.10000002 sec
D11	0.03000000 sec
TD0	1
SFO1	150.9656784 MHz
NUC1	13C
P1	10.00 usec
PLW1	88.38300323 W
SFO2	600.3224013 MHz
NUC2	1H
CPDPRG[2	waltz16
PCPD2	80.00 usec
PLW2	21.85499954 W
PLW12	0.48474309 W
PLW13	0.24295320 W

F2 - Processing parameters

SI		32768
SF		150.9505840 MHz
WDW		EM
SSB	0	
LB		1.00 Hz
GB	0	
PC		1.40
